# Mechanistic insights into antiretroviral drug‐induced liver injury

**DOI:** 10.1002/prp2.598

**Published:** 2020-07-08

**Authors:** Jamie N. Pillaye, Mohlopheni J. Marakalala, Nonhlanhla Khumalo, Wendy Spearman, Hlumani Ndlovu

**Affiliations:** ^1^ Division of Chemical and System Biology Department of Integrative Biomedical Sciences Faculty of Health Sciences University of Cape Town Cape Town South Africa; ^2^ Africa Health Research Institute Durban KwaZulu Natal South Africa; ^3^ Division of Infection and Immunity University College London London UK; ^4^ Hair and Skin Research Lab Division of Dermatology Department of Medicine Groote Schuur Hospital and University of Cape Town Cape Town South Africa; ^5^ Division of Hepatology Department of Medicine Groote Schuur Hospital and University of Cape Town Cape Town South Africa

**Keywords:** antiretroviral therapy, diagnosis, drug‐induced liver injury, human immunodeficiency virus, mechanisms

## Abstract

All classes of antiretroviral therapy (ART) have been implicated to induce adverse drug reactions such drug‐induced liver injury (DILI) and immune‐mediated adverse reactions in Human Immunodeficiency Virus (HIV) infected individuals. Patients that develop adverse drug reactions tend to have prolonged stays in hospital and may require to change to alternative regimens if reactions persist upon rechallenge or if rechallenge is contraindicated due to severity of the adverse reaction. Diagnosis of DILI remains a huge obstacle that delays timely interventions, since it is still based largely on exclusion of other causes. There is an urgent need to develop robust diagnostic and predictive biomarkers that could be used alongside the available tools (biopsy, imaging, and serological tests for liver enzymes) to give a specific diagnosis of DILI. Crucial to this is also achieving consensus in the definition of DILI so that robust studies can be undertaken. Importantly, it is crucial that we gain deeper insights into the mechanism of DILI so that patients can receive appropriate management. In general, it has been demonstrated that the mechanism of ART‐induced liver injury is driven by four main mechanisms: mitochondrial toxicity, metabolic host‐mediated injury, immune reconstitution, and hypersensitivity reactions. The focus of this review is to discuss the type and phenotypes of DILI that are caused by the first line ART regimens. Furthermore, we will summarize recent studies that have elucidated the cellular and molecular mechanisms of DILI both in vivo and in vitro.

## 1 INTRODUCTION

Acquired Immune Deficiency Syndrome (AIDS), caused by Human Immunodeficiency Virus (HIV), is a highly communicable disease that has placed a tremendous burden on national healthcare systems globally but specifically in sub‐Saharan Africa. The disease was estimated to affect 37.9 million people worldwide in 2018, directly causing approximately 770 000 deaths.^1^ The prevalence of HIV infections is the highest in Eastern and Southern Africa where an estimated 20.6 million people are living with HIV/AIDS. Co‐infection with opportunistic infections, predominantly tuberculosis (TB), have further increased the morbidity and mortality rate of HIV‐infected individuals. In 2018, 1.5 million individuals died from TB, of which 400 000 were HIV positive, making TB the top killer of people living with HIV/AIDS.

The successful roll out of antiretroviral therapy (ART) has reduced the risk of early mortality due to opportunistic infections and improved both the prognosis as well as the quality of life for HIV‐infected patients. There was an estimated 24.5 million people receiving ART worldwide in 2018, a significant increase from 7.7 million people that were accessing ART in 2010.^1^ This jump in the number of people accessing ART seems to have been propelled by the implementation of the “test and treat” policy of the World Health Organisation (WHO) in 2015. Consequently, there has also been a 33% decline in AIDS‐related deaths since 2010,^1^ indicating the success of the current efforts to treat and control the disease.

Unfortunately, some patients receiving ART may develop severe adverse drug reactions such as drug‐induced liver injury (DILI). The reported incidence of ART‐induced liver injury varies greatly due a number of factors such as a criteria used to define the severity of hepatotoxicity, geographical location, prevalence of chronic viral hepatitis infections, genetic heterogeneity in populations, frequency of liver enzyme determinations, medication prescribing patterns, and other exogenous exposures.^2^, ^3^, ^4^ Hence, the reported incidence of ART‐induced DILI ranges from 8%‐23% in HIV‐infected patients and up to 30% of these patients may require change of regimen or the discontinuation of therapy.^4^, ^5^, ^6^, ^7^ DILI is a common cause of prolonged hospitalization in HIV‐infected patients and in severe cases may be fatal. Discontinuation of the offending drug can lead to treatment failure, and the potential emergence of drug resistant pathogens. Studies have shown that efavirenz (EFV), a first line ART drug is a key driver of DILI in HIV‐infected individuals taking the first line EFV/Tenofovir disoproxil fumarate/Emtricitabine fixed‐dose combination. The reported risk factors for DILI are female gender, young age, and high CD4 counts.^8^, ^9^

In this review, we will discuss the different patterns and severity of DILI and describe the types and phenotypes of DILI caused by ART in HIV‐infected individuals. We will also provide a summary of the studies that have analysed the molecular mechanisms of DILI with a particular focus on cellular toxicity, immunological responses, and biochemical pathways that have been implicated to drive hepatotoxicity of first line ART. It is important to note that the WHO recently introduced new guidelines that recommended the use of dolutegravir alongside two nucleoside reverse transcriptase inhibitors (NRTIs) as a first line regimen.^10^ Although there were some initial concerns about the safety of dolutegravir, particularly in pregnant women,^11^ subsequent studies have shown it to be safe.^12^, ^13^ The effects of this new regimen will not be the focus of this review.

### 1.1 Patterns and severity of DILI

There has been great variability in the criteria used in clinical studies to define DILI and/or the severity of hepatotoxicity. Some studies have defined DILI as elevations in liver enzymes (alanine aminotransferase [ALT] and aspartate aminotransferase [AST]) two times the upper limit of normal (ULN),^14^, ^15^ while others used an absolute threshold of liver enzymes regardless of the baseline levels.^16^, ^17^ Efforts have been made to achieve consensus in the definition and grading of DILI.^18^, ^19^, ^20^, ^21^ The clinical presentations of DILI have been classified biochemically and clinically into three main categories based on *R* value (*R* = (ALT value ÷ ALT ULN) ÷ (ALP value ÷ ALP ULN)). DILI is categorized as hepatocellular when *R* is more than 5 (*R* > 5), cholestatic when *R* is less than 2 (*R* < 2), and mixed when *R* is more than 2 but less than 5 (2 < *R* < 5).^19^, ^20^, ^22^ It is important to mention that ARTs are mainly associated with hepatocellular DILI that usually arise within one year of starting the offending therapy.

The DILI Expert Working Group and AIDS Clinical Trial Group graded DILI severity into four grades based on baseline ALT or alkaline phosphatase (ALP) below the ULN.^18^, ^21^ Grade 1 (mild) is defined by 1.25‐2.5x ULN ALT or ALP; Grade 2 (moderate) is characterized by 2.6‐5.0x ULN ALT or ALP; Grade 3 (severe) defined by 5.1‐10x ULN ALT or ALP; and Grade 4 (severe) is defined by greater than 10x ULN ALT or ALP, or death, or transplantation due to DILI.^18^, ^21^ Incidences of DILI as well as the associated side effects vary between individuals due to their genetic profile and whether they are consuming other drugs. Hence, for the effective management of DILI, a more personalized treatment approach may be required.

All classes of ARTs cause DILI in HIV‐infected patients; however, some classes are more toxic than others. Table 1 provides a list of ARTs that are known to cause adverse reactions in HIV positive individuals. Non‐nucleoside reverse transcriptase inhibitors (NNRTI) such as nevirapine (NVP) have been shown to induce DILI in 12% of the patients, which is three times more than that caused by EFV.^23^ Similar results were reported by Sanne and colleagues, who found that patients treated with a regimen containing NVP had a DILI incidence rate of 17%, while those treated with EFV did not develop DILI.^24^ This study recruited HIV‐infected men and nonpregnant women who were ART naïve, had plasma HIV‐1 RNA levels greater than 5000 copies/mL and CD4^+^ cell count that was greater than 200 cells/mm^3^.^24^ Importantly, 385 patients were enrolled in the NVP arm while the EFV arm had only 83 patients, hence, introducing a possible bias and skewing of the data. Therefore, these data need to be interpreted with more caution. Although these studies indicate a higher risk of liver toxicity for patients receiving NVP compared to EFV, EFV has been shown to be toxic in both treatment naïve and experienced patients.^9^, ^25^, ^26^

**TABLE 1:** Summary of the antiretroviral drugs with a known adverse reaction in HIV positive patients

Drug	Class	Adverse reaction	References
Nevirapine	NNRTI	DILI, hypersensitivity	4, 5, 6
Efavirenz	NNRTI	DILI, hypersensitivity	4, 5, 6
Zidovudine	NRTI	DILI	27
Stavudine	NRTI	DILI	27
Didanosine	NRTI	DILI	27
Abacavir	NRTI	Hypersensitivity	75, 80, 81
Indinavir	PI	DILI	27, 29
Tipranivir	PI	DILI	27, 29
Aplaviran	CCR5 antagonist	DILI	30

Abbreviations: DILI, drug‐induced liver injury; HIV, Human Immunodeficiency Virus; NNRTI, non‐nucleoside reverse transcriptase inhibitors; NRTI, nucleoside reverse transcriptase inhibitor; PI, protease inhibitor.

Nucleoside reverse transcriptase inhibitors, protease inhibitors (PIs), and fusion inhibitors have also been reported to cause DILI in HIV‐infected patients. NRTIs such as zidovudine, stavudine, and didanosine caused moderate to severe DILI while emtricitabine, abacavir, and tenofovir induce minor elevations in liver enzymes.^27^ PIs have been reported to cause DILI in 1%‐9.5% of the patients, with a few patients that have been reported to develop severe elevation in liver enzymes.^27^ There have been a few case reports of liver toxicity with indinavir and tipranavir, particularly in patients with underlying diseases such as cirrhosis and chronic hepatitis C infection.^28^, ^29^ The majority of PI’s reported to cause DILI are not constituents of the fixed‐dose drug regimen that is composed of EFV, tenofovir and emtricitabine. GlaxoSmithKline halted a Phase 2b and 3 clinical trials for Aplaviran, a CCR5 antagonist, after four men from approximately 300 patients developed severe liver disease.^30^ The other CC5R antagonists, maraviroc and vicriviroc, appear to be safe. Enfuvirtide, the only approved fusion inhibitor, appears to be well‐tolerated and safe.^31^ Similarly, integrase inhibitors such as MK‐0518^32^ and dolutegravir have been demonstrated to be safe and tolerable in treatment naïve and experienced adults and adolescents.^33^, ^34^, ^35^, ^36^ Moreover, a recent observational study conducted in Botswana showed that dolutegravir‐based ART regimen was safe for pregnant women.^11^

### 1.2 Types and major phenotypes of drug induced liver injury

Drug induced liver injury (DILI) has been mainly classified as either direct or idiosyncratic; however, indirect injury is emerging as a third class.^37^, ^38^ Direct hepatotoxicity is attributed to the drugs or metabolites that are intrinsically toxic to the liver, biliary epithelial cells, and the liver vasculature. It is common, predictable, dose‐dependent, and reproducible in animal models.^37^ Direct hepatotoxicity occurs rapidly, with a latency period of one and five days after intake of high therapeutic doses of a drug. This type of injury is associated with elevations in liver enzymes such as ALT and ALP without the accompanying hyperbilirubinemia.^39^ The elevations in liver enzymes can subside when the offending drug is stopped or dose is reduced—but in some cases can spontaneously resolve, a phenomenon referred to as adaptation.^40^

#### 1.2.1 Direct hepatotoxicity

Clinically, the most common feature of direct hepatotoxicity is acute hepatic necrosis, and severe cases present with acute hepatic failure characterized by encephalopathy and coagulopathy.^20^, ^41^ Interestingly, histological examination depicts a centrilobular and panlobular necrosis with little inflammation. It is interesting to note that NNRTIs such as NVP and EFV have been reported to cause similar pathological features with some distinctive features such as inflammation with lymphocytes, eosinophils, and plasma cells. For instance, NVP has been shown to be more toxic causing portal tract expansion and severe inflammatory reactions with eosinophils infiltrating the parenchyma.^42^ Analysis of liver biopsies obtained from patients that received a regimen containing EFV revealed that submassive necrosis was associated with significant morbidity and mortality.^8^, ^25^ Submassive necrosis is characterized by zonal/pan‐zonal necrosis with an “immune‐allergic” pattern and recruitment of inflammatory cells, such lymphocytes, plasma cells, and eosinophils.^25^ A study by Elsharkawy and colleagues identified a first adult case of EFV induced acute liver failure that required liver transplantation within 5 months after initiating therapy.^43^ Interestingly, this 43‐year‐old woman was a slow drug metabolizer, which contributed to the severe clinical presentation.^43^ The first generation NRTI’s such as zidovudine, stavudine, and didanosine have been reported to cause nodular regenerative hyperplasia, a noncirrhotic portal hypertension.^44^ Finally, didanosine has been shown to induce hyperlactatemia and lactic acidosis that has an onset within months of initiating therapy in HIV‐infected individuals ^45^

#### 1.2.2 Idiosyncratic hepatotoxicity

Idiosyncratic hepatotoxicity occurs in rare cases (typically 1 to 2000 and 1 to 100 000 patient‐exposures) and is usually caused by drugs with little or no intrinsic toxicity to the liver.^46^, ^47^ It is unpredictable, dose‐independent, and unreproducible in animal models. Idiosyncratic injury is classified into three categories based on the *R* value; hepatocellular, cholestatic, and mixed.^20^ Idiosyncratic liver injury frequently manifests as mixed injury with some hepatocellular hepatitis.^46^, ^48^, ^49^ Drugs such as isoniazid, nitrofurantoin, and diclofenac have been incriminated as the common causes of idiosyncratic hepatocellular injury.^48^, ^49^, ^50^, ^51^ Idiosyncratic drug‐induced injury may be associated with an immune‐allergic reaction such as rash, fever, and eosinophilia—all typical signs of hypersensitivity.^48^, ^49^, ^52^ NVP and EFV have been reported to be the most common causes of skin rashes in patients taking first line ART regimens and have an incidence rate of 10%‐17%.^53^, ^54^, ^55^ NNRTIs induce a mild macular, maculopapular, or erythematous rash and a severe rash characterized with blistering, moist desquamation, and ulceration.^53^ Numerous reviews have been written on hypersensitivity reactions to ARTs in HIV‐infected individuals.^19^, ^56^, ^57^, ^58^

#### 1.2.3 Indirect hepatotoxicity

Indirect hepatotoxicity is caused by the action of the drug in triggering a new liver condition or worsening an underlying condition such as viral hepatitis than its intrinsic toxicity or idiosyncratic properties.^38^ Indirect toxicity presents with the characteristics of the underlying condition or predisposition. ART‐mediated immune reconstitution inflammatory syndrome has been reported to trigger flares of liver enzymes, spontaneous seroconversion, and exacerbation of hepatitis caused by Hepatitis B and C virus (HBV and HCV).^59^ In fact, numerous studies have reported that HIV‐infected patients with an underlying chronic HBV or HCV may develop severe toxicity to ART and need closer monitoring.^60^, ^61^, ^62^, ^63^, ^64^, ^65^, ^66^, ^67^

### 1.3 The role of genetic polymorphisms in ART‐induced liver injury

#### 1.3.1 Drug metabolizing enzymes

Genetic variations in drug metabolizing enzymes have been associated with risk of adverse drug reaction in patients taking ART (Table 2). A study by Phillips and colleagues found an association between *CYP2B6* slow metabolizer genotype with NVP driven hypersensitivity reactions (rash) but not with hepatotoxicity.^68^ This was further demonstrated in a recent study by de Almeida and colleagues who analysed the drug metabolism gene polymorphisms in a Brazilian cohort that received an EFV‐based regimen and found that the *CYP2B6* slow metabolizer genotype was associated with an increased risk of EFV adverse reaction.^69^ They also showed that CNS adverse effects were associated with *CYP3A4* rs4646437 genotype.^69^, ^70^ In a Thai cohort, the *CYP2B6* haplotype *6/*6 was identified in 8.2% of the HIV‐infected patients that received an EFV‐based regimen and it was associated with high ALP and total bilirubin, suggesting that this genotype may correlate with an increased risk of hepatotoxicity in patients taking an EFV‐based ART regimen.^71^ Similar findings were observed in a prospective cohort Ethiopian study that revealed an association between the *CYP2B6* *6/*6 genotype with ART‐induced liver injury and high plasma concentrations of EFV.^72^ Therefore, it may be necessary to screen individuals for the *CYP2B6* genotypes so they could be given therapy that would have minimal adverse effects to encourage compliance treatment or know the most likely CYP2B6 genotypes within a population to decide on pragmatic therapy.

**TABLE 2:** Genetic variations in Cytochrome P450 and human leucocyte antigen genes that are associated with ART induced adverse reactions

Gene	Genotype/allele	Drugs	Adverse reaction	Reference
*CYP2B6*	6/6	Nevirapine, efavirenz	DILI, hypersensitivity	69, 71, 105
*HLA‐DRB1*	0101	Nevirapine	DILI, hypersensitivity	78, 83
*HLA‐DRB1*	0102	Nevirapine	DILI	79
*HLA‐B*	5701	Abacavir	Hypersensitivity	75, 80, 81
*HLA‐B*	3505	Nevirapine	Hypersensitivity	84, 85
*HLA‐B*	5801	Nevirapine	DILI	79
*HLA‐C*	0401	Nevirapine	Hypersensitivity	85, 86

Abbreviations: ART, antiretroviral therapy; DILI, drug‐induced liver injury.

#### 1.3.2 Cytokines

The immune response plays a critical role in driving some of the adverse drug reactions that are associated with ART in HIV‐infected individuals. The severity of DILI is influenced by the presence of toxic molecules and a critical counterbalance of cytokines such as tumor necrosis factor‐alpha, interleukin‐1beta (IL‐1β), IL‐10 and IL‐1RN.^68^ In fact, some studies have associated polymorphisms in cytokine genes with the development of ART‐associated hepatotoxicity^73^ and hypersensitivity.^74^ A recent study by Singh and colleagues investigated *IL‐1RN* (VNTR) and *IL‐1β* (‐511C/T) polymorphisms in 34 HIV‐positive patients with ARV hepatotoxicity, 128 HIV‐positive patients without hepatotoxicity, and 152 healthy controls using PCR and PCR‐RFLP. They found that *IL‐1RN* 2/2 and 1/3 genotypes were highly represented in patients with hepatotoxicity to a NVP based ARV regimen.^75^, ^76^, ^77^ Moreover, *IL‐1β*‐511CT and ‐511TT genotypes enhanced the risk of hepatotoxicity in patients taking the NVP based regimen. Finally, a study by Asensi and colleagues showed that *IL‐1β*3954T allele is associated with lipodystrophy syndrome in patients on ART.^78^, ^79^

#### 1.3.3 Human leucocyte antigen

The interactions between the T cells and professional antigen presenting cells via the class I or class II human leucocyte antigen (HLA) plays a key role in development of adverse drug reactions. In fact, HLA‐B*5701 polymorphism has been linked with abacavir hypersensitivity in HIV‐positive individuals,^75^, ^80^, ^81^, ^82^ while the HLA‐DRB1*0101^78^ and HLA‐B*3505^83^, ^84^, ^85^ was associated with increased risk of developing NVP‐driven hypersensitivity reactions.^79^, ^83^, ^85^, ^86^, ^87^ Moreover, HLA‐C*0401 allele is associated with hypersensitivity to NVP and is carried across most ethnicities.^85^, ^86^, ^87^ HLA‐DRB*0101 has been associated with a risk of developing NVP induced hepatitis across ethnicities.^83^, ^85^ A study by Phillips and colleagues that recruited 385 South African participants who initiated a NVP containing regimen showed that HLA‐B*5801 and HLA‐DRB1*0102 were associated with hepatotoxicity.^79^ All these alleles and the adverse reaction that are triggered by ART are summarized in Table 2. Hence, HLA typing applied as a precision medicine tool could significantly reduce a risk of adverse reactions to ART. In fact, this has been implemented for abacavir hypersensitivity and has been hailed as a huge success as revealed in a recent meta‐analysis by Stainsby and colleagues.^82^

### 1.4 Molecular mechanisms of ART‐driven hepatotoxicity

#### 1.4.1 Effect of drugs on cellular morphology

The molecular mechanism of hepatotoxicity induced by ART seems to be multifactorial. A number of studies have reported that EFV causes cellular damage indicated by changes in the cells morphology, endoplasmic reticulum (ER), and mitochondrial integrity.^88^, ^89^, ^90^ A study by Apostolova and colleagues showed that Hep3B cells treated with EFV displayed a concentration dependent mitochondrial dysfunction and damage compared to control cells that contained smooth mitochondria with complete membranes and distinct and well‐formed cristae.^88^, ^89^ Hep3B cells are an immortalized human hepatoma cell line that expresses little or no drug metabolizing enzymes that are required for drug metabolism and toxicity studies.^88^, ^89^ Moreover, Hep3B cells exposed to EFV displayed an abnormal cellular morphology with a swollen phenotype.^89^ A higher concentration of EFV induced severe damage depicted by visible alterations of the mitochondrial outer membranes, reduction in the number of mitochondrial cristae and change in surface structure.^89^

Mitochondrial toxicity is a common pathway linked with the use of NRTIs in HIV positive individuals.^91^ The mitochondria is an essential organelle that is critical for energy production, metabolism of glucose and fats, and production of reactive oxygen species (ROS) that could be deleterious to the cells. A study by de Mendoza and colleagues showed that HIV‐positive patients taking a stavudine‐containing regimen had significantly reduced mitochondrial DNA copy number compared to HIV‐positive patients that were on other regimens, and this was associated with elevated lactate levels.^92^ Mitochondrial toxicity can manifest in patients taking NRTI drugs as nonspecific symptoms to lactic acidosis syndrome that may be accompanied by fulminant hepatic failure.^92^, ^93^ Moreover, serum analysis can reveal modest elevations in liver enzymes, with AST greater than ALT. Unfortunately, the mortality rate is high in patients who develop lactic acidosis syndrome, but administration of specific therapy with cofactors can lower the mortality rate.^94^

#### 1.4.2 Bioenergetics and signaling pathways

**FIGURE 1:**
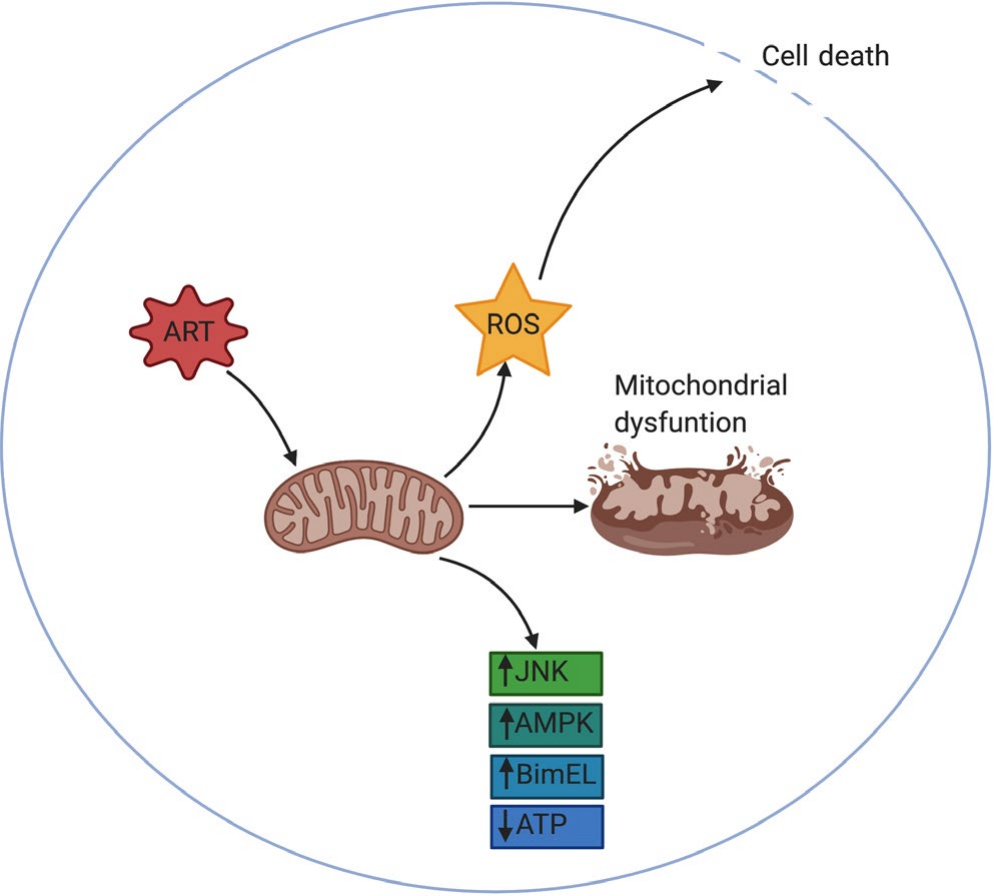
Antiretroviral therapy caused mitochondirial dysfunction and increased ROS production in vitro. Antiretroviral therapy, particularly efavirenz has been implicated in causing mitochondrial dysfunction, increased production of reactive oxygen species (ROS), leading to cell death. Moreover, drugs induced increased expression of JNK, AMPK and BimEL. AMPK, adenosine monophosphate‐activated protein kinase; BimEL, BIM‐extra‐long; JNK, Jun N‐terminal kinases

Research has implicated the involvement of respiratory chain proteins,^88^, ^95^ abnormal Jun N‐terminal kinases (JNK) and BIM‐extra‐long (BimEL) signaling pathways in the development of ARV induced cytotoxicity (Figure 1).^96^ A study by Perier and colleagues showed that some ART drugs inhibit complex I (NADH: ubiquinone oxidoreductase) of the electron transport chain (ETC), resulting in defects in mitochondrial bioenergetics due to reduced flow of electrons and impairment of oxidative phosphorylation,^97^ especially in tissues with high energy demand such as the liver. The inhibition of complex I by EFV has been shown to result in the accumulation of lipids in the cytoplasm of both human hepatic tissue as well as Hep3B cells.^98^ As expected, the inhibition of complex I impairs the respiratory chain resulting in reduced levels of adenosine triphosphate (ATP), increased production of ROS, and continuous activation of adenosine monophosphate‐activated protein kinase (AMPK), which is the major regulator of cellular bioenergetics^98^, ^99^, ^100^ (Figure 1). Interestingly, methylene blue, an alternative electron carrier which can bypass the proximal ETC, can prevent the energy crisis and protect against lethal cell injury associated with the mitochondria targeting drugs.^96^ These results are compatible with the concept that underlying silent mitochondrial dysfunction may be a susceptibility factor contributing to DILI.^96^

Efavirenz is known to increase the activity of AMPK, which results in increased production of ROS and possibly initiates cell death.^101^? In fact, treating primary mouse hepatocytes with EFV triggered cell death that was associated with complex I inhibition, peroxynitrite formation and ATP depletion.^101^ Interestingly, deletion of *sirt3* gene encoding Sirtuin‐3, a major mitochondrial NAD^+^‐dependent deacetylase in mice (Sirt3 knockout mice) protected hepatocytes from EFV‐induced cytotoxicity compared to cells from wild‐type mice.^101^ Therefore, induction of stress responses, particularly the production of ROS and peroxynitrite formation is implicated in causing cell death in EFV treated cells.

Another interesting biochemical family of serine/threonine kinase is the JNK. These are important regulators of cellular stress responses including modulation of cell death.^101^ Studies have reported that EFV treated primary human hepatocytes undergo apoptotic cell death that requires JNK activation and BimEL expression (Figure 1).^101^ Moreover, these effects can be recapitulated via treatment of hepatocytes using synthetic 8‐hydroxy EFV, which is the primary metabolite of EFV.^102^ The inhibition of cytochrome P450‐mediated metabolism markedly decreased the toxicity of EFV in human hepatocytes measured by cell death, caspase‐3 activation and ROS formation.^102^ The potential role of JNK and BimEL signaling pathways and its involvement in EFV‐mediated hepatocyte death makes it a possible target for design and development of drugs to mitigate EFV‐induced hepatotoxicity.

A study by Lee et al showed that in murine hepatocytes, the high concentrations of isoniazid alone did not cause acute cell injury.^102^ However, there was evidence that acute exposure to isoniazid caused mitochondrial abnormalities, specifically changes in the oxygen consumption rate.^98^ When Rotenone, a potent complex II inhibitor was added with INH, there was a noticeable decrease in hepatotoxicity.^98^ Interestingly, a study by Lee and colleagues also found that neither EFV nor isoniazid were able to induce cell death alone.^103^ However, exposure to a combination of EFV and isoniazid resulted in increased oxidative and nitrosative stress, leading to the formation of membrane permeability transition, and ultimately necrotic cell death.^73^ These data demonstrated that a combination of drugs causing hepatotoxicity can induce mitochondrial dysfunction, leading to necrotic cell death.

#### 1.4.3 Effects of drugs on the ER and autophagy

Careful analysis of cellular morphology by transmission electron microscopy (TEM) revealed autophagic degradation of the mitochondria and the ER appeared to be wrapped around the mitochondria, possibly in order to generate a membrane that would later be incorporated into the autophagic vacuoles.^95^ Further evaluation of cellular morphology in Hep3B cells and primary human hepatocytes exposed to EFV by TEM revealed a change in ER morphology and the presence of granular deposits.^90^ The granular deposits may represent an accumulation of unfolded proteins. Therefore, it appears that cellular damage is one of the key mechanisms responsible for hepatotoxicity induced by anti‐HIV drugs, particularly EFV.

At cellular level, treating Hep3B cells with lower concentrations of EFV has been shown to induce autophagy as evidenced by the presence of autophagic vacuoles and expression of specific autophagic protein markers such as microtubule‐associated protein 1A/1B light chain 3 and Beclin‐1.^89^ Autophagy is an essential lysosomal pathway that is required for maintaining cell function and survival through degradation of proteins, cellular components, and organelles.^89^, ^104^ However, exposing Hep3B cells to higher concentrations of EFV resulted in blockade of autophagic flux which induced autophagic stress, and ultimately promoted severe cellular damage.^89^ Counterintuitively, specific inhibition of autophagy with 3‐methyladenine (3MA) in Hep3B cells treated with EFV had the deleterious effect on cell survival/proliferation by promoting apoptosis, which suggests that autophagy may act as an adaptive mechanism of cell survival.^89^ More work is required to elucidate the role of autophagy at cellular level, especially given that ARV regimens are changing with the introduction of new drugs to fixed does combinations.

## 2 CONCLUSION

Adverse drug reactions including DILI present an enormous challenge for the treatment and management of HIV‐infected patients. This is further compounded by other complexities such as the emergence of drug resistance, introduction of new multidrug regimens, and co‐morbidities like TB and viral hepatitis. One cannot underscore a need for a breakthrough in diagnosis of DILI and how this will aid in the management of the adverse reactions so that patients can adhere to their treatment regimen. A good example to cite here is the success that has been achieved by taking a precision medicine approach in dealing with the abacavir hypersensitivity and implementing testing for HLA‐B57:01 genotype prior to prescribing the treatment.^82^ More pharmacogenetic and pharmacokinetic studies need to be conducted to identify other critical genotypes that may be contributing to the advent of DILI in this vulnerable group of patients, particularly in light of the changing regimens and doses.

Efforts have been made over the years to achieve consensus in the definition of DILI.^16^, ^18^, ^20^, ^21^ This will facilitate easier interpretation and comparison of future studies whilst also enabling researchers to design robust studies in light of the changing multidrug regimens for HIV. Gaining mechanistic insights into how ART causes DILI is essential for the identification of possible diagnostic targets and also potential therapeutic candidates that could be targeted to ameliorate the adverse reactions. Genetic variations in genes encoding for drug metabolizing enzymes (*CYPs*) have been associated with hepatotoxicity and hypersensitivity reactions to some ART.^78^, ^79^, ^83^, ^84^, ^86^ The mitochondrial toxicity of ART is not unexpected given the fact that the mitochondria is an energy hub of a cell and plays a critical role in the detoxification of xenobiotics. Recent mechanistic studies have demonstrated that EFV can inhibit oxidative phosphorylation,^96^, ^98^ while triggering ER stress^88^ and activation of AMPK,^97^, ^99^, ^101^ JNK,^101^ and BimEL^101^ signaling pathways. Although we have gained some crucial insights into the biochemical underpinnings of cytotoxicity caused by the drugs, more work is still necessary to comprehensively unravel the critical players and lead to the identification of possible drug targets.

The immune system plays a critical role in augmenting and mediating some of the adverse reactions such as DILI and hypersensitivity reactions. Studies have incriminated polymorphisms in cytokine genes^68^, ^73^, ^74^ and the HLA^75^, ^76^, ^77^, ^79^, ^80^, ^81^, ^86^, ^87^ that predispose‐HIV‐infected individuals to adverse reactions to ART. There is a paucity of studies dissecting the contribution of different immune subsets in the pathogenesis of DILI in HIV‐infected patients that are on therapy. Beyond identifying the various subtypes, it would also be necessary to analyse their activation states and key regulatory circuits. Technologies such as single cell RNA sequencing could be deployed to answer these lingering scientific questions.

## CONFLICT OF INTEREST

The authors have no conflict of interest to declare.

## AUTHOR CONTRIBUTIONS

JP, MJM, NK, WCS, and HN: conception and design of manuscript; JP and HN: manuscript writing; JP, MJM, NK, WCS,and HN: reviewed and edited the manuscript; HN and MJM: provided financial and administrative support; HN: final approval of the manuscript.
